# Curcumin attenuates acute inflammatory injury by inhibiting the TLR4/MyD88/NF-κB signaling pathway in experimental traumatic brain injury

**DOI:** 10.1186/1742-2094-11-59

**Published:** 2014-03-27

**Authors:** Hai-tao Zhu, Chen Bian, Ji-chao Yuan, Wei-hua Chu, Xin Xiang, Fei Chen, Cheng-shi Wang, Hua Feng, Jiang-kai Lin

**Affiliations:** 1 Department of Neurosurgery, Southwest Hospital, Third Military Medical University, 30 Gaotanyan Street, Chongqing 400038, China; 2 Department of Neurobiology, Third Military Medical University, 30 Gaotanyan Street, Chongqing 400038, China

**Keywords:** Toll-like receptor 4, Curcumin, Traumatic brain injury, Inflammation

## Abstract

**Background:**

Traumatic brain injury (TBI) initiates a neuroinflammatory cascade that contributes to substantial neuronal damage and behavioral impairment, and Toll-like receptor 4 (TLR4) is an important mediator of thiscascade. In the current study, we tested the hypothesis that curcumin, a phytochemical compound with potent anti-inflammatory properties that is extracted from the rhizome *Curcuma longa*, alleviates acute inflammatory injury mediated by TLR4 following TBI.

**Methods:**

Neurological function, brain water content and cytokine levels were tested in TLR4^-/-^ mice subjected to weight-drop contusion injury. Wild-type (WT) mice were injected intraperitoneally with different concentrations of curcumin or vehicle 15 minutes after TBI. At 24 hours post-injury, the activation of microglia/macrophages and TLR4 was detected by immunohistochemistry; neuronal apoptosis was measured by FJB and TUNEL staining; cytokines were assayed by ELISA; and TLR4, MyD88 and NF-κB levels were measured by Western blotting. *In vitro*, a co-culture system comprised of microglia and neurons was treated with curcumin following lipopolysaccharide (LPS) stimulation. TLR4 expression and morphological activation in microglia and morphological damage to neurons were detected by immunohistochemistry 24 hours post-stimulation.

**Results:**

The protein expression of TLR4 in pericontusional tissue reached a maximum at 24 hours post-TBI. Compared with WT mice, TLR4^-/-^ mice showed attenuated functional impairment, brain edema and cytokine release post-TBI. In addition to improvement in the above aspects, 100 mg/kg curcumin treatment post-TBI significantly reduced the number of TLR4-positive microglia/macrophages as well as inflammatory mediator release and neuronal apoptosis in WT mice. Furthermore, Western blot analysis indicated that the levels of TLR4 and its known downstream effectors (MyD88, and NF-κB) were also decreased after curcumin treatment. Similar outcomes were observed in the microglia and neuron co-culture following treatment with curcumin after LPS stimulation. LPS increased TLR4 immunoreactivity and morphological activation in microglia and increased neuronal apoptosis, whereas curcumin normalized this upregulation. The increased protein levels of TLR4, MyD88 and NF-κB in microglia were attenuated by curcumin treatment.

**Conclusions:**

Our results suggest that post-injury, curcumin administration may improve patient outcome by reducing acute activation of microglia/macrophages and neuronal apoptosis through a mechanism involving the TLR4/MyD88/NF-κB signaling pathway in microglia/macrophages in TBI.

## Introduction

Traumatic brain injury (TBI) is defined as damage to the brain resulting from an external mechanical force, which can lead to temporary or permanent impairment of cognitive, physical and psychosocial functions [1]. It is the leading cause of death and disability for people under the age of 45 years. Ten million deaths and/or hospitalizations annually are directly attributable to TBI, and an estimated 57 million living people worldwide have experienced such brain injury [2].

It is well known that TBI is a highly complex disorder that is caused by both primary and secondary brain injury mechanisms. Secondary brain injury, which results from delayed neurochemical, metabolic and cellular changes, can evolve over hours to days after the initial traumatic insult and cause progressive white and gray matter damage. A complex series of sterile inflammatory responses play an important role in secondary brain injury following TBI [3,4]. However, a detailed understanding of the effect of innate immunity after TBI remains limited at present. The innate immune system recognizes different pathogens via highly conserved microbial motifs, namely pathogen-associated molecular patterns (PAMPs), through pathogen-recognition receptors (PRRs) [5]. Toll-like receptors (TLRs) are a family of PRRs that recognize conserved microbial motifs in molecules such as bacterial lipopolysaccharide (LPS), peptidoglycan, flagellin, and double- and single-stranded viral RNAs. Recently, it has been shown that TLRs become activated in response to endogenous ligands released during tissue injury, such as the degradation products of macromolecules, heat shock proteins and intracellular components of ruptured cells, known as damage-associated molecular patterns (DAMPs) [6]. Microglia, the principal cells involved in the innate immune response in the CNS, express robust levels of TLR1-9 [7]. Among these TLRs, TLR4 has been shown to play an important role in initiating the inflammatory response following stroke or head trauma [8-10]. Furthermore, myeloid differentiation factor 88 (MyD88), a critical adapter protein for TLR4, leads to the activation of downstream NF-κB and the subsequent production of proinflammatory cytokines implicated in neurotoxicity [11,12].

Curcumin, a major component extracted from the rhizome *Curcuma longa*, has been consumed by humans as a curry spice for centuries. It has been extensively studied for its wide range of biological activities, including anti-inflammatory, anti-oxidant, anti-infection and anti-tumor properties [13]. *In vivo*, curcumin has been found to cross the blood-brain barrier and maintain high biological activity [14], and it has been proposed for the treatment of various neuroinflammatory and neurodegenerative conditions in the CNS. Recent studies have demonstrated that curcumin is a highly pleiotropic molecule that interacts with numerous molecular targets [15]. Thus far, although a few studies indicate that curcumin can attenuate cerebral edema, promote membrane and energy homeostasis and influence synaptic plasticity following TBI [16-19], the modulatory effects of curcumin on the inflammatory response after TBI remain largely unknown. Recently, *in vitro*, curcumin has been shown to inhibit the homodimerization of TLR4, which is required for the activation of downstream signaling pathways [20,21]. The presumption that curcumin can attenuate inflammatory injury via the TLR4 pathway has since been tested in some models of injury [22-25], but it remains unknown whether exogenous curcumin can modulate TBI through the TLR4/MyD88/NF-κB signaling pathway. We designed this study to investigate the importance of TLR4 in initiating the acute inflammatory response following TBI, which contributes to neuronal damage and behavioral impairment, and to confirm the hypothesis that curcumin attenuates acute inflammatory damage by modulating the TLR4/MyD88/NF-κB signaling pathway in microglia/macrophages during experimental TBI.

## Materials and methods

### Animals

Adult male C57BL/6 mice (8 to 10 weeks, 20 to 25 g) were provided by the Animal Center of Third Military Medical University. Transgenic TLR4^-/-^ mice (8 to 10 weeks, 20 to 22 g) were purchased from Jackson Laboratories (Bar Harbor, ME, USA) and were backcrossed to a C57BL/6 background more than eight times. All experiments were conducted in accordance with animal care guidelines approved by the Animal Ethics Committee of the Third Military Medical University. The animals were housed in temperature- and humidity-controlled animal quarters with a 12-hour light/dark cycle and water and food provided *ad libitum*. Mice were treated with an intraperitoneal injection of curcumin (Sigma, St. Louis, MO, USA) dissolved in 100 μL of dimethyl sulfoxide (DMSO) (50, 100, 200 mg/kg) or equal volumes of vehicle 15 minutes post-TBI. In our experiment, each test was performed independently for either three times (three mice per group) or twice (six mice per group).

### Experimental traumatic brain injury model in mice

TBI was induced using a Feeney weight-drop contusion model with slight modifications [26]. Mice were anesthetized with intraperitoneal chloral hydrate (40 mg/kg) and placed in a stereotaxic frame, and a 4 mm craniotomy was performed over the right parietal cortex, centered on the coronal suture and 3 mm lateral to the sagittal suture. Considerable care was taken to avoid injury to the underlying dura. A weight-drop device was placed over the dura. An impact transducer (foot plate) was adjusted to stop at a depth of 2.5 mm below the dura. Then, one 18 g weight was dropped from 10 cm above the dura through a guide tube onto the foot plate. Body temperature was maintained with an overhead heating lamp during the experiments. Dural tears were not repaired and the bone flap was not re-inserted. If the animals demonstrated dural tears or excessive bleeding, they were excluded. After injury, the skin was closed tightly. To maintain normal body temperature during surgery and recovery, the mice were maintained with isothermic (37°C) heating. Mice in the sham-operation group were subjected to the same surgical procedure, including craniotomy, but received no cortical impact.

### Neurological function evaluation

Behavioral testing was performed one day after TBI using the mNSS (modified Neurological Severity Score) assessment. The mNSS is a composite of motor, sensory, reflex and balance tests [27]. One point was scored for the inability to perform each test or for the lack of a tested reflex; thus, the higher the score was, the more severe the injury. Neurological function was graded on a scale of 0 to 18 (normal score, 0; maximal deficit score, 18).

### Brain water content

Twenty-four hours post-injury, brain edema was determined using the wet/dry method:

Percent brain water = [(Wet weight–Dry weight)/Wet weight] · 100% [28]

The brains from mice in each treatment group were rapidly removed from the skull, and the brains were separated bilaterally, weighed and then placed in an oven for 72 hours at 100°C. The brains were then reweighed to obtain dry weight content.

### Cortical neuronal cultures

Cortical cells were prepared from embryonic day 15 pregnant mice. Briefly, embryos were removed, the cerebral cortex was dissected, and meninges were stripped in Ca^2+^/Mg^2+^-free Hank’s balanced saline solution (HBSS) solution. Tissues were then digested in 0.125% trypsin for 15 minutes and dispersed through the narrowed bore of a fire-polished Pasteur pipette and passed through a 40 μm cell strainer. Cells were distributed in a poly-L-lysine-coated (Sigma) culture plate containing 0.5 mL of neurobasal medium with 2% B27 supplement (Invitrogen, Carlsbad, CA, USA). The culture density was 5 × 10^5^ cells/mL. Cultures were maintained at 37°C in a humidified incubator with 5% CO_2_/95% room air. All transwell co-culture experiments were performed with neurons that had been in culture for seven days.

### Microglial cultures

The cortices of the cerebral hemispheres of one-day-old post-natal mice were dissected and digested with 0.25% trypsin. After centrifugation for five minutes at 300 × g, the cortical cells were seeded in DMEM-F12 with 10% FBS on a 25 cm^2^ flask at a density of 3 × 10^5^ cells/mL and cultured at 37°C in humidified 5% CO_2_/95% air. The medium was replaced every four to five days, and confluency was achieved after 14 days *in vitro*. Microglial cells were obtained by shaking the flasks overnight. Floating cells were pelleted and subcultured at 3 × 10^5^ cells/mL in glial-conditioned medium on poly-L-lysine pre-coated transwell inserts. Cell purity was determined by immunohistochemical staining with microglia-specific antibodies for CD11b, and purity was determined to be > 95%.

### Transwell co-cultures

Transwell co-cultures were performed as previously described [29]. Microglia were plated onto the top side of the transwell inserts (0.4 μm pore size polyester membrane precoated with poly-L-lysine; Corning, NY, USA) at the cell density described above. The transwells were positioned approximately 2 mm above the neuron-enriched culture plate, and the microglia grown on the transwells were separated from the neurons by the permeable transwell membrane. Then, 1 μg/ml LPS (Sigma, St. Louis, MO, USA), curcumin, LPS plus curcumin or DMSO (Sigma, St. Louis, MO, USA) as a solvent control was added to the media below the transwells.

### Cytotoxicity assay

Cell viability was evaluated by the 3-(4,5-dimethylthiazol-2-yl)-2,5-diphenyltetrazolium bromide (MTT) reduction assay. In brief, neurons (5 × 10^5^ cells/mL) and microglia (3 × 10^5^ cells/mL) were seeded in the transwell system, as described above, and treated with various concentrations of curcumin. After 24 hours of incubation, the medium was removed. The neurons and microglia were separated and then incubated with 0.5 mg/mL MTT solution. After incubation for three hours at 37°C in 5% CO_2_, the supernatant was removed, and the formation of formazan crystals was measured at 490 nm with a microplate reader.

### Immunofluorescence

Mice were perfused transcardially with saline, followed by 4% paraformaldehyde under deep anesthesia (100 mg/kg sodium pentobarbital) and their brains sectioned at a 20 μm thickness using a cryostat. The sections were blocked in 5% normal donkey serum diluted in PBS for one hour at room temperature and then incubated overnight at 4°C with mouse anti-TLR4 or rat anti-CD11b as the primary antibody. Donkey anti-mouse Alexa-Fluor 568 and donkey anti-rat Alexa-Fluor 488 were used as secondary fluorescent probes. The sections were viewed by confocal microscopy (LSM780, Zeiss, Jena, Germany) and analyzed as individual images for TLR4 and CD11b co-expression. Immunostained sections were quantitatively characterized by digital image analysis using Image Pro-Plus 6.0 software (Media Cybernetics, Silver Spring, MD, USA). TLR4 was quantified as the average number of positive cells per field. A negative (no antibody) control was included.

Cell cultures were fixed for 30 minutes in 4% paraformaldehyde. Cells were blocked with 1% bovine serum for one hour. Cultures were incubated overnight at 4°C with primary antibody. Microglia were incubated with mouse anti-TLR4 (1:400, ab22048;Abcam, Cambridge, MA, USA) or rat anti-CD11b (1:200, ab8878;Abcam, Cambridge, MA, USA). Neurons were incubated with mouse anti-tubulin (1:400, MAB1637; Millipore, Billerica, MA, USA). Alexa 488 and Alexa 568 secondary fluorescent antibodies (1:400, Invitrogen, Carlsbad, CA, USA) were used for one hour at 37°C, and the nuclei were stained with 4',6-diamidino-2-phenylindole(DAPI) for ten minutes. The cells were observed by confocal microscopy. The images were analyzed individually to evaluate TLR4 and CD11b co-expression, and the immunofluorescence intensity of TLR4 per field was determined using Image Pro-Plus 6.0 software(Media Cybernetics, Silver Spring, MD, USA). A negative (no antibody) control was included.

### Western blot analysis

Protein was extracted from the cortex surrounding the injured area and cultured microglia or neurons using a protein extraction kit (P0027, Beyotime Biotech,Jiangsu, China). The lysate was separated by centrifugation at 12,000 × g at 4°C for 15 minutes, and the supernatant was collected. The protein concentration was determined using a BCA assay kit (P0010, Beyotime Biotech, Jiangsu, China). Nuclear protein (for NF-κB p65) and other cytoplasmic proteins were diluted in the loading buffer and subjected to sodium dodecyl sulfate polyacrylamidegel electrophoresis(SDS-PAGE) followed by transfer to PVDF membranes. The membrane was blocked with 5% freshly prepared milk-TBST for two hours at room temperature and then incubated overnight at 4°C with the following primary antibodies: mouse anti-TLR4 (1:400, ab22048;Abcam, Cambridge, MA, USA), rabbit anti-MyD88 (1:400, ab2064;Abcam, Cambridge, MA, USA), mouse anti-NF-κB (1:400, sc-8008; Santa Cruz Biotechnology Inc., Santa Cruz, CA, USA), rabbit anti-cleaved caspase-3 (1:400, 9661; CST, Danvers, MA, USA), rabbit anti-IκB-α (1:400, sc-371; Santa Cruz Biotechnology Inc., Santa Cruz, CA, USA), mouse anti-phosphorylated-IκB-α (1:400, sc-8404; Santa Cruz Biotechnology Inc., Santa Cruz, CA, USA, USA) and β-actin (1:1,000, AA128;Beyotime Biotech, Jiangsu, China). After the membrane was washed in TBST, it was incubated in the appropriate AP-conjugated secondary antibody (diluted 1:2,000 in secondary antibody dilution buffer) for one hour at 37°C. Protein bands were visualized by nickel-intensified DAB solution according to previous reports [30]. The β-actin antibody was used as an internal standard. The optical densities of the detected proteins were obtained using Image Pro-Plus software 6.0 (Media Cybernetics, Silver Spring, MD, USA).

### Enzyme-linked immunosorbent assay (ELISA)

Brain tissue in the cerebral cortex around the injured area was collected and homogenized. The homogenates were centrifuged at 4°C at 12,000 × g for 15 minutes, and supernatants were collected carefully and evaluated in duplicate using IL-1β, IL-6, TNF-α, MCP (monocyte chemoattractant protein)-1 and RANTES (regulated upon activation, normal T cell expressed and secreted) assay kits (R&D Systems, Minneapolis, MN, US), in accordance with the manufacturer’s guidelines. Tissue cytokine concentrations are expressed as picograms per milligram of protein.

Cell culture supernatants were carefully collected at 24 hours after stimulation with LPS and centrifuged at 4°C at 12,000 × g for 15 minutes. Cytokine concentrations were evaluated using protein assay kits (R&D Systems, Minneapolis, MN, US), in accordance with the manufacturer’s guidelines. Cell cytokine concentrations are expressed as picograms per milliliter.

### FJB histochemistry

Fluoro-Jade B (FJB) is a polyanionic fluorescein derivative that binds with high sensitivity and specificity to degenerating neurons. FJB staining of brain sections was performed as previously described with slight modifications [31]. Briefly, selected sections were first incubated in a solution of 1% NaOH in 80% ethanol for five minutes and then rehydrated in 70% ethanol and distilled water for two minutes each. The sections were then incubated in 0.06% KMnO_4_ for ten minutes, rinsed in distilled water for two minutes and incubated in a 0.0004% solution of FJB (Chemicon, Temecula, CA, USA) for 20 minutes. Sections were observed and photographed under a confocal microscope.

### TUNEL staining

The TUNEL assay was performed using a commercial kit that labels DNA strand breaks with fluorescein isothiocyanate (FITC; *In Situ* Cell Death Detection Kit, Roche Molecular Biochemicals, Mannheim, Germany). Selected sections were pretreated with 20 mg/mL proteinase-K in 10 mM Tris-HCl at 37°C for 15 minutes. These sections were then rinsed in PBS and incubated in 0.3% hydrogen peroxide dissolved in anhydrous methanol for ten minutes. The sections were then incubated in 0.1% sodium citrate and 0.1% Triton X-100 solution for two minutes at 2 to 8°C. After several washes with PBS, sections were incubated with 50 μL of TUNEL reaction mixture with terminal deoxynucleotidyltransferase (TdT) for 60 minutes at 37°C under humidified conditions, and neuronal nuclei were stained with DAPI. Each section was observed and photographed under a confocal microscope. Negative controls were obtained by omitting the TdT enzyme.

### Statistical analysis

All data are presented as the mean ± SD. SPSS 11.5 was used for statistical analysis of the data. Two-way repeated-measures ANOVAs with LSD *posthoc* tests were used to determine statistical significance between behavioral measures. One-way ANOVAs with the appropriate LSD *posthoc* tests were used to compare experimental groups. For all analyses, *P* < 0.05 was considered significant.

## Results

### Time-dependent protein expression of TLR4

**Figure 1:**
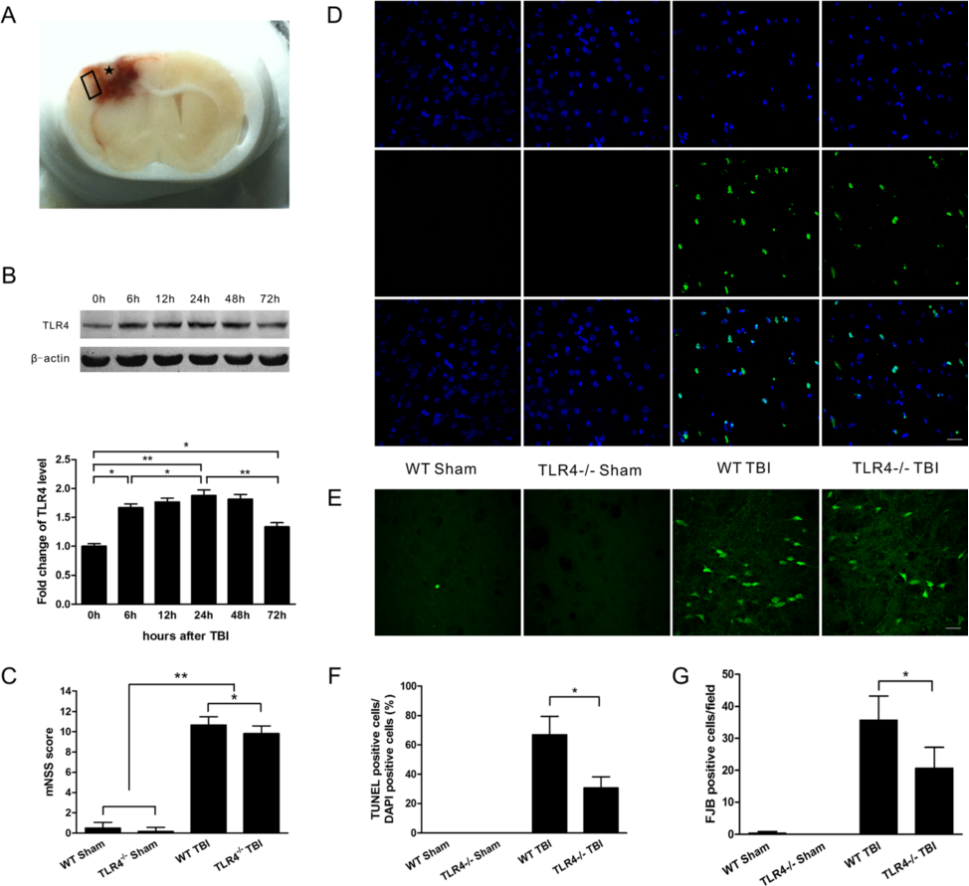
**TLR4-/- mice displayed attenuations in the neurological deficit and cell death. (A)** A coronal brain slice showing an obvious cavity (marked by an asterisk) in the injured cortex. The tissue examined in the experiment is marked by a box. **(B)** Time-dependent protein expression of TLR4 in the injured tissue. **(C)** The neurological deficit score of TLR4^-/-^ mice was significantly lower than that of wild-type (WT) mice at 24 hours post-trauma. **(D)** Representative TUNEL-stained and 4',6-diamidino-2-phenylindole (DAPI)-stained brain sections at 24 hours post-trauma. **(E)** Representative Fluoro-Jade B (FJB-stained) brain sections at 24 hours post-trauma. **(F)** Quantification analysis indicated that TLR4^-/-^ mice had significantly fewer TUNEL-positive cells in the pericontusional tissue than WT mice post-trauma. The percentage of TUNEL-positive cells is expressed as the number of TUNEL-stained nuclei divided by the total number of DAPI-stained nuclei. **(G)** Quantification showed that TLR4^-/-^ mice had significantly fewer degenerating neurons than WT mice in the pericontusional tissue. The total number of FJB-positive cells is expressed as the mean number per field of view. Values (mean ± SD) are representative of three independent experiments (n = 3 *P < 0.05, **P < 0.01. Bar = 20 μm.

A coronal brain slice showed an obvious cavity in the injured cortex, which was surrounded by hemorrhage. The tissue examined in the experiment is indicated by a box in the figure (Figure 1A). Basal TLR4 expression was low in the sham control brains. The expression of TLR4 was significantly increased in the injured tissue at six hours post-trauma (*P* < 0.05) and reached a maximum at 24 hours (*P* < 0.01); thereafter, it decreased but remained high through 72 hours post-TBI (*P* < 0.05) (Figure 1B).

### TLR4 deficiency attenuated neurological deficit, cerebral edema, cytokine release and cell death post-trauma

**Figure 2:**
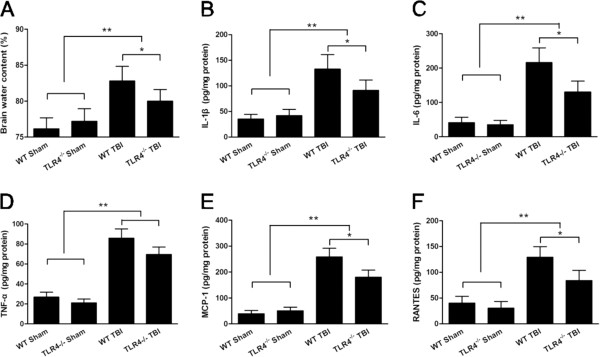
**TLR4**^**-/-**^**mice displayed attenuated brain edema and neuroinflammation post-trauma. (A)** TLR4^-/-^ mice displayed decreased brain water content compared with WT mice. ELISA showed a change in the release of IL-1β, IL-6, TNF-α, MCP-1 and RANTES **(B, C, D, E, F)** in TLR4^-/-^ mice brain tissue 24 hours post-trauma. Values (mean ± SD) are representative of three independent experiments (n = 3 mice/group). *P < 0.05, **P < 0.01.

To confirm the role of TLR4 in TBI, TLR4^-/-^ mice were used to investigate cerebral edema, neurological function impairment and the release of cytokines post-trauma in comparison with WT mice. The neurological deficit score of TLR4^-/-^ mice was significantly lower than that of WT mice at 24 hours post-trauma (*P* < 0.05, Figure 1C). The brain water content of TLR4^-/-^ mice was also significantly lower than that of WT mice at 24 hours post-trauma (*P* < 0.05, Figure 2A). Moreover, the IL-1β, IL-6, MCP-1 and RANTES protein concentrations in the injured brain tissue, as determined by ELISA, were also significantly decreased in TLR4^-/-^ mice compared with WT mice (*P* < 0.05, Figure 2B, C, E, F), but the TNF-α concentration was not significantly different between TLR4^-/-^ and WT mice (*P* > 0.05, Figure 2D). In addition, neuronal and apoptotic cell death were alleviated in TLR4^-/-^ mice. Both FJB-positive cells with neuronal morphology and TUNEL-positive cells were evident 24 hours post-trauma in the pericontusional tissue (Figure 1D, E). The number of TUNEL-positive cells increased dramatically around the injured tissue in the TBI groups at 24 hours post-trauma. However, significantly fewer TUNEL-positive cells were found in TLR4^-/-^ mice than in WT mice (*P* < 0.05, Figure 1F). Furthermore, TLR4^-/-^ mice also had significantly fewer FJB-positive neurons in the pericontusional tissue when compared with WT mice (*P* < 0.05, Figure 1G).

### Downregulation of TLR4 expression by curcumin treatment post-trauma

**Figure 3:**
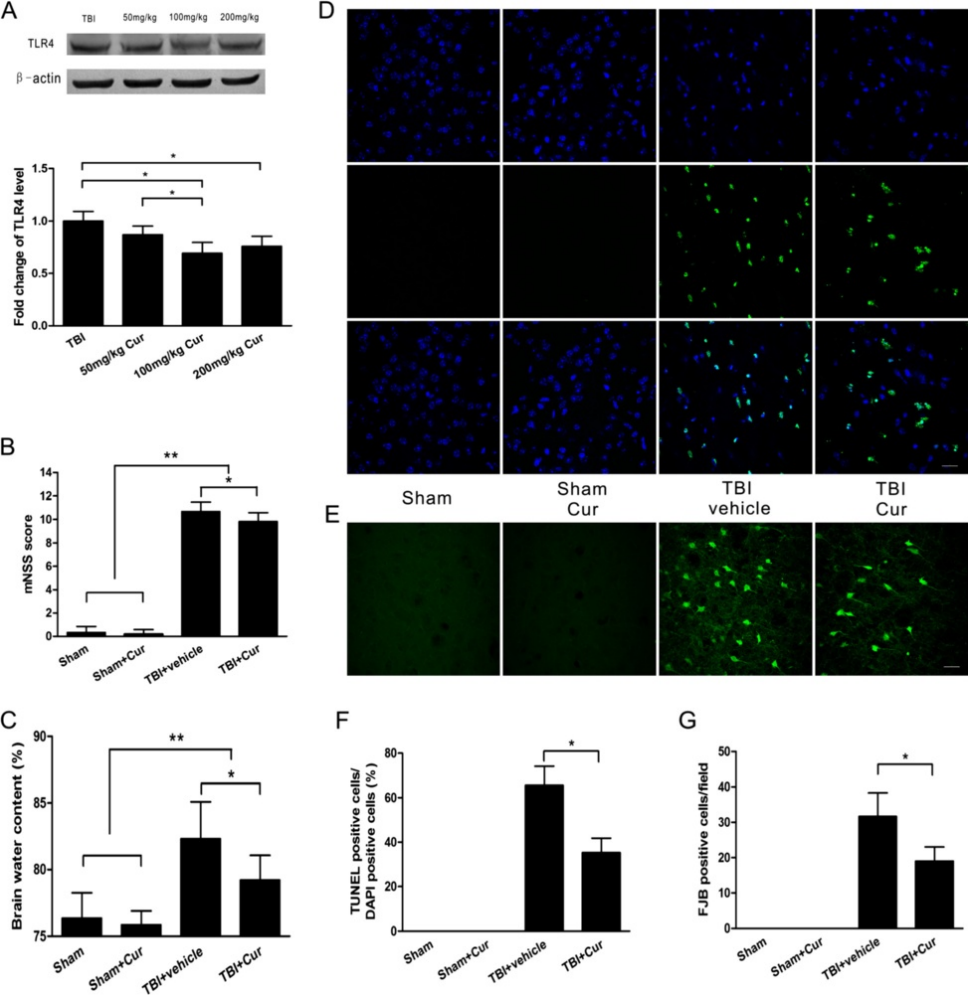
**Curcumin attenuated brain injury post-trauma. (A)** The effect of different concentrations of curcumin on TLR4 expression in injured tissue at 24 hours post-trauma. Curcumin treatment decreased the neurological deficit scores **(B)** and brain water content **(C). (D)** Representative TUNEL-stained and 4',6-diamidino-2-phenylindole (DAPI)-stained brain sections at 24 hours post-trauma. **(E)** Representative Fluoro-Jade B (FJB)-stained brain sections at 24 hours post-trauma. **(F)** Quantification analysis indicated that curcumin-treated mice had significantly fewer TUNEL-positive cells in the pericontusional tissue than vehicle-treated mice. The percentage of TUNEL-positive cells is expressed as the number of TUNEL-stained nuclei divided by the total number of DAPI-stained nuclei. **(G)** Quantification showed that curcumin-treated mice had significantly fewer degenerating neurons than vehicle-treated mice in the pericontusional tissue. The total number of FJB-positive cells is expressed as the mean number per field of view. Values (mean ± SD) are representative of two independent experiments (n = 6 mice/group). *P < 0.05, **P < 0.01. Bar = 20 μm.

Because TLR4 deficiency resulted in neuroprotection, we next examined the effects of curcumin on TLR4 protein expression. We administered different concentrations of curcumin (50, 100, or 200 mg/kg) to mice fifteen minutes post-TBI and examined TLR4 expression 24 hours post-trauma. The administration of 50 mg/kg curcumin did not significantly reduce TLR4 expression compared with TBI alone (*P* > 0.05, Figure 3A). In contrast, 100 mg/kg or 200 mg/kg curcumin significantly reduced TLR4 expression (*P* < 0.01 versus TBI alone), but TLR4 expression did not significantly differ between these two groups (*P* > 0.05). Accordingly, 100 mg/kg was selected due to the dramatic reduction of TLR4 expression and the relatively low concentration of curcumin.

### Neuroprotection of curcumin post-trauma

Curcumin attenuated cerebral edema and improved neurological function following TBI. The neurological deficit scores were significantly lower in curcumin-treated mice than in vehicle-treated mice at 24 hours post-trauma (*P* < 0.05, Figure 3B). Brain water content was significantly decreased in curcumin-treated mice when compared with vehicle-treated mice at 24 hours post-trauma (*P* < 0.05, Figure 3C). In addition, curcumin reduced neuronal and apoptotic cell death. Both FJB-positive cells with neuronal morphology and TUNEL-positive cells were evident 24 hours post-trauma in the pericontusional tissue (Figure 3D, E). The number of TUNEL-positive cells was increased dramatically around the injured tissue in the TBI groups at 24 hours post-trauma. Significantly fewer TUNEL-positive cells were found in curcumin-treated mice than in vehicle-treated mice (*P* < 0.05, Figure 3F). Furthermore, curcumin-treated mice also had significantly fewer FJB-positive neurons in the pericontusional tissue than did the vehicle-treated group (*P* < 0.05, Figure 3G).

### Curcumin inhibited the activation of TLR4-positive microglia/macrophages and inflammatory mediator release in injured tissue

**Figure 4:**
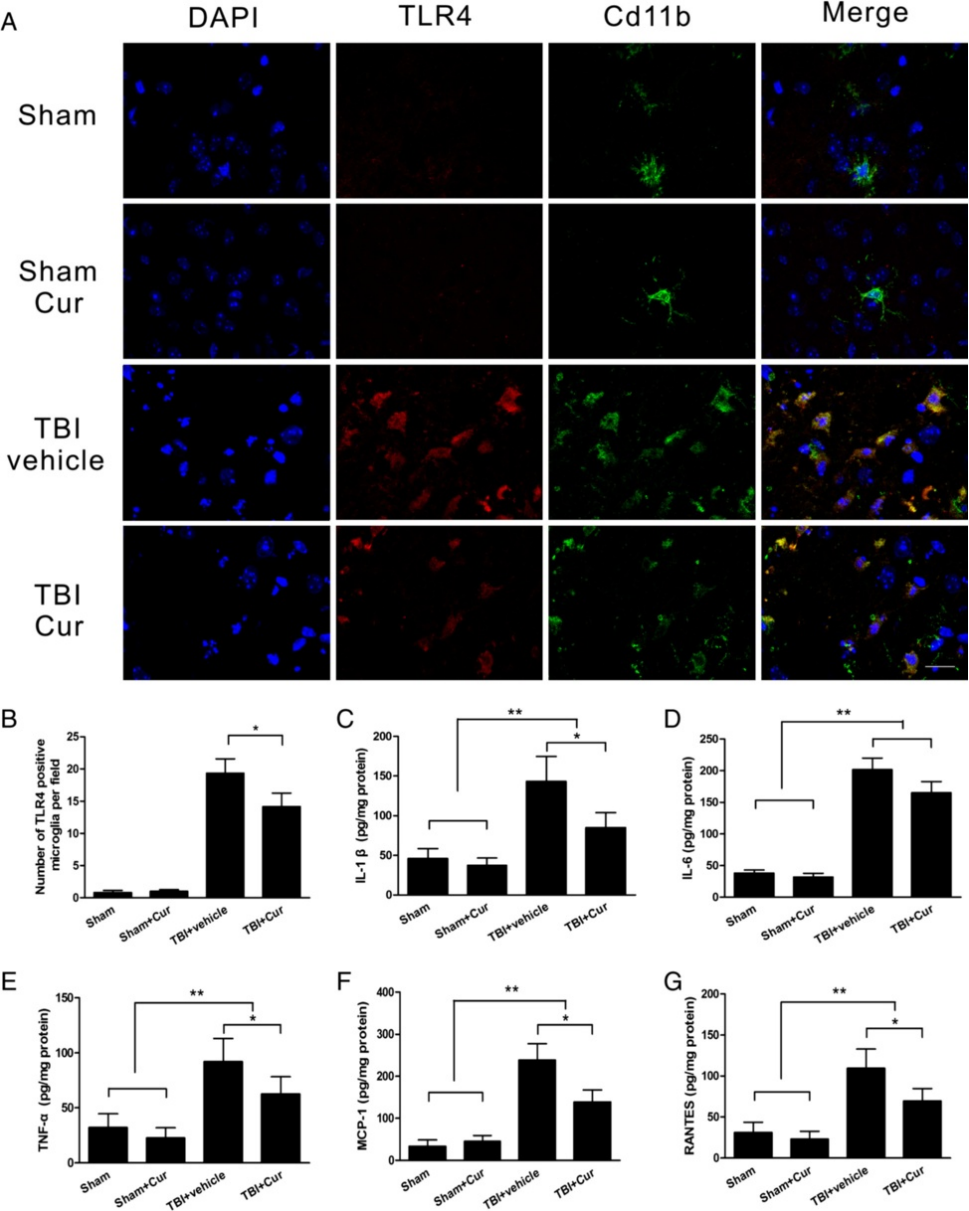
**Curcumin decreased neuroinflammation and the activation of CD11b-positive cells co-labeled with TLR4 post-trauma. (A)** Representative CD11b-positive cells co-labeled with TLR4 in the pericontusional tissue at 24 hours post-trauma. **(B)** Quantification showed that curcumin-treated mice had significantly fewer CD11b-positive cells co-labeled with TLR4 in the pericontusional tissue than vehicle-treated mice. The total number of CD11b-positive cells co-labeled with TLR4 is expressed as the mean number per field of view. ELISA showed that curcumin treatment resulted in a change in the release of IL-1β, IL-6, TNF-α, MCP-1 and RANTES **(C, D, E, F, G)** at 24 hours post-trauma. Values (mean ± SD) are representative of two independent experiments (n = 6 mice/group). *P < 0.05, **P < 0.01. Bar = 20 μm.

In the pericontusional tissue of sham control mice, a few quiescent microglia with small cell bodies and fine, ramified processes were observed 24 hours post-trauma. Few or no TLR4-positive microglia were detected. However, many activated TLR4-positive microglia/macrophages (CD11b-positive cells) with large cell bodies and thickened, short processes were observed post-trauma. These microglia/macrophages exhibited robust TLR4 immunoreactivity (Figure 4A). The administration of 100 mg/kg curcumin inhibited the increase in TLR4-positive microglia/macrophages post-trauma (*P* < 0.05, Figure 4B), although microglia/macrophages still exhibited reactive morphology. Moreover, the concentrations of inflammatory mediators (IL-1β, IL-6, TNF-α, MCP-1 and RANTES) in the injured brain tissue, determined using ELISA, were significantly increased in the two TBI groups when compared with the two sham groups (*P* < 0.01), and these mediators were all dramatically decreased in curcumin-treated mice when compared with vehicle-treated mice, with the exception of IL-6 (*P* < 0.05, Figure 4C-G).

### Curcumin suppressed protein expression in the TLR4/MyD88/NF-κB signaling pathway *in vivo*

**Figure 5:**
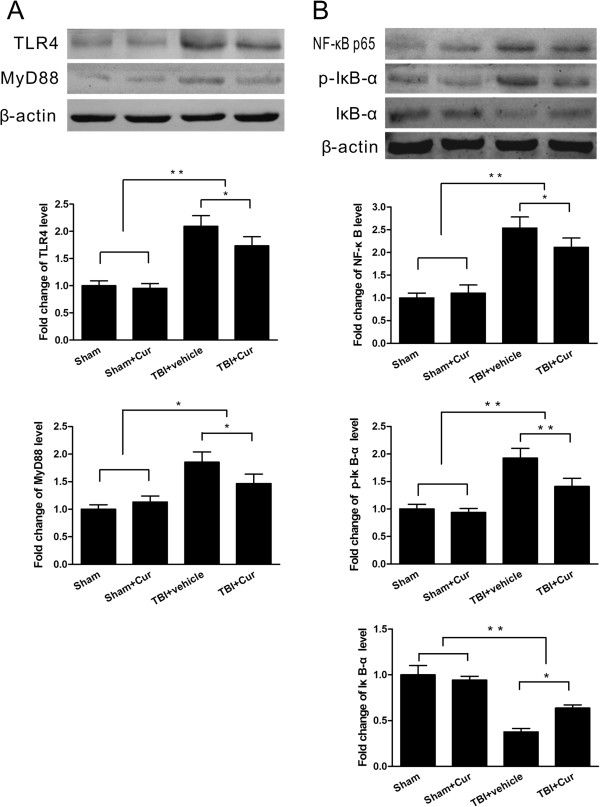
**Curcumin suppressed TLR4/MyD88/NF-**κ**B signaling pathway protein expression *in vivo*. (A)** TLR4 and MyD88 protein expression in the injured tissue was significantly lower in curcumin-treated mice than in vehicle-treated mice at 24 hours post-trauma. **(B)** NF-κB p65 and p-IκB-α protein expression in the injured tissue was also significantly lower in curcumin-treated mice than in vehicle-treated mice at 24 hours post-trauma. In contrast, IκB-α protein expression was significantly higher in curcumin-treated mice than in vehicle-treated mice post-trauma. Values (mean ± SD) are representative of three independent experiments (n = 3 mice/group). *P < 0.05, **P < 0.01.

Western blotting showed that TLR4 and MyD88 protein expression in the injured tissue was increased dramatically in the TBI groups when compared with the sham control groups (*P* < 0.01) and that it was significantly lower in curcumin-treated mice than in vehicle-treated mice at 24 hours post-trauma (*P* < 0.05, Figure 5A). NF-κB p65 and p-IκB-α protein expression in the injured tissue was also increased dramatically in the TBI groups but was significantly decreased in curcumin-treated mice compared to the vehicle-treated mice at 24 hours post-trauma (*P* < 0.05, Figure 5B). In contrast, IκB-α protein expression was decreased in the TBI groups but was significantly increased in curcumin-treated mice when compared with vehicle-treated mice at 24 hours post-trauma (*P* < 0.05, Figure 5B).

### Curcumin reduced neuronal damage induced by LPS *in vitro*

**Figure 6:**
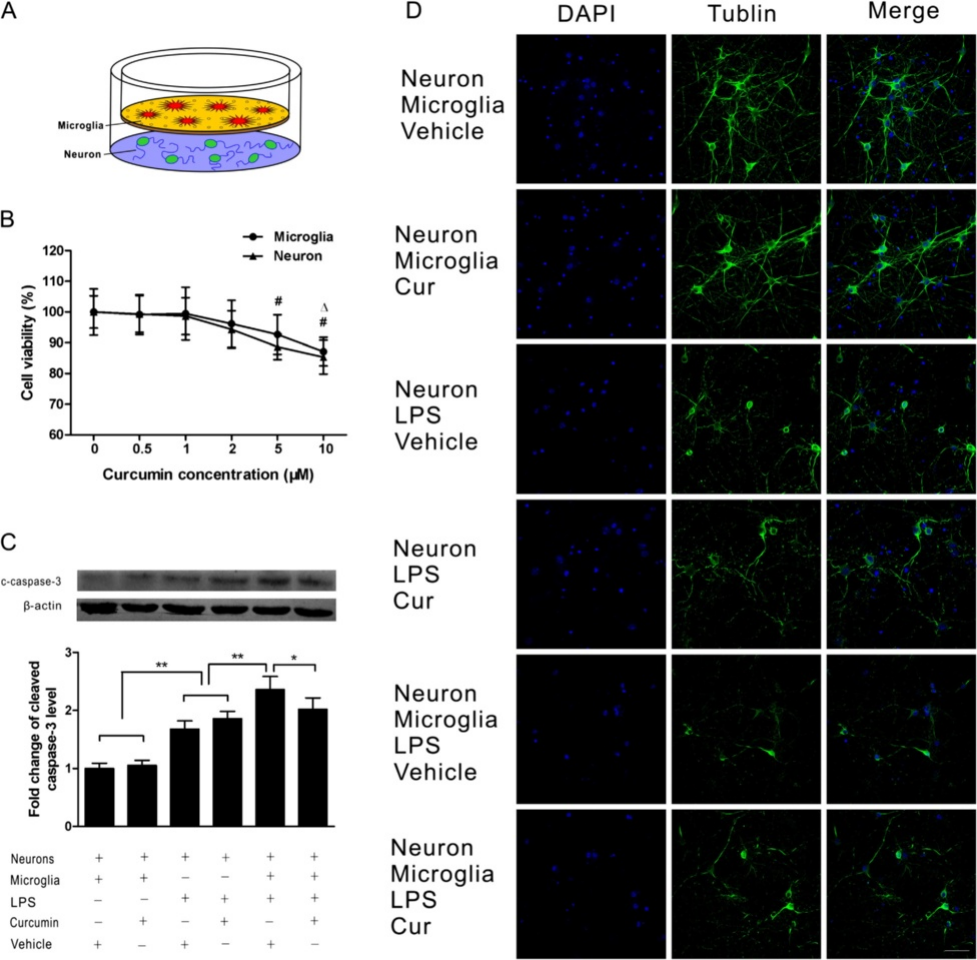
**Curcumin attenuated the neuronal damage induced by lipopolysaccharide (LPS) in the transwell co-culture of neurons and microglia. (A)** In a transwell system, microglia and neurons were cultured together, as shown. **(B)** Cell viability following the administration of different concentrations of curcumin. **(C)** Protein level of cleaved caspase-3 in neurons in co-culture and single-culture systems. Curcumin significantly reduced cleaved caspase-3 in the co-culture system after LPS stimulation. **(D)** Morphological changes of neurons in the single-culture and co-culture systems. Curcumin attenuated morphological damage in the co-culture system after LPS stimulation but did not have an effect in the single-culture system. Values (mean ± SD) are representative of three independent experiments. ^*^P < 0.05, compared with the no-curcumin treatment group for microglial viability; ^#^*P* < 0.05, compared with the no-curcumin treatment group for neuronal viability; *P < 0.05, **P < 0.01. Bar = 50 μm.

To directly observe the interaction of microglia and neurons, we used a transwell co-culture system including primary neurons and microglia and stimulated the cells with LPS. Microglia were plated onto the transparent polyester membrane of the transwell inserts, and neurons were placed on the wells below the polyester membrane; as a result, the microglia grown on the transwells were separated from the neuron-enriched cultures by the permeable transwell membrane (Figure 6A). To determine the optimal concentration of curcumin for cell co-culture, 0.5, 1, 2, 5 and 10 μM were applied separately. The administration of 10 μM curcumin significantly reduced microglial viability compared with the no-curcumin control (*P* < 0.05), whereas the cell viability in the 0.5, 1, 2 and 5 μM curcumin treatment groups did not significantly differ from that in the control group (*p* > 0.05, Figure 6B). However, 5 and 10 μM curcumin both significantly reduced neuronal viability when compared with the no-curcumin control (*P* < 0.05, Figure 6B). Accordingly, 2 μM was chosen as the optimal concentration for the transwell co-culture system.

We then examined neuronal damage under various conditions. The protein levels of cleaved caspase-3 in neurons were significantly increased 24 hours after LPS stimulation (*P* < 0.01), and the protein level in co-cultured neurons was significantly higher than that in the single-culture group (*P* < 0.05). In the co-culture groups, curcumin treatment after LPS administration significantly decreased the upregulation of cleaved caspase-3 (*P* < 0.05). In contrast, in the single-culture groups, curcumin treatment after LPS stimulation did not significantly decrease the upregulation of cleaved caspase-3 (*P* > 0.05, Figure 6C). Similar results were observed using immunofluorescence. At 24 hours after LPS administration, many neuronal bodies and processes were destroyed or no longer evident, and more serious neuronal damage was observed in the co-culture group than in the single-culture group. However, when the cells were treated with curcumin after LPS stimulation, less serious neuronal damage was observed in the co-culture groups, whereas no marked change in neuronal damage was observed in the single-culture groups (Figure 6D).

### Curcumin attenuated the microglial activation and inflammatory mediator release induced by LPS *in vitro*

**Figure 7:**
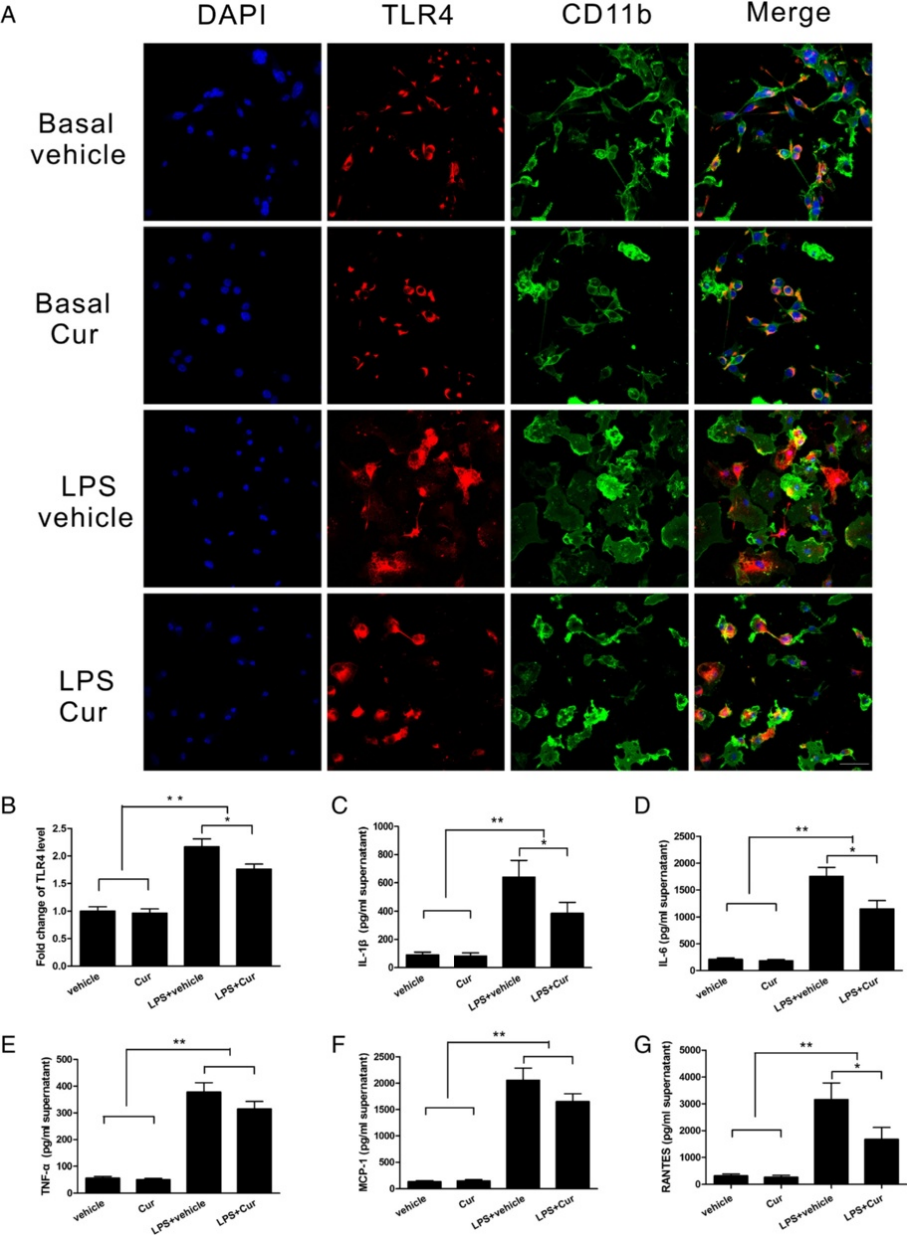
**Curcumin reduced microglial activation and inflammatory mediator release induced by lipopolysaccharide (LPS) in the transwell co-culture of neurons and microglia. (A)** Colocalization of CD11b and TLR4 was evident. Treatment with curcumin after LPS stimulation resulted in a less reactive state of the microglia, as shown. **(B)** TLR4 immunofluorescence intensity in microglia was also reduced after curcumin treatment. ELISA showed that curcumin-treated cells had a change in the release of IL-1β, IL-6, TNF-α, MCP-1 and RANTES **(C, D, E, F, G)** at 24 hours after LPS administration. Values (mean ± SD) are representative of three independent experiments. *P < 0.05, **P < 0.01. Bar = 50 μm.

In the transwell co-culture experiments, LPS stimulation induced a reactive state in the microglia, which was demonstrated by a larger cell body and thickened, shorter processes, and these microglia also showed robust TLR4 immunofluorescence intensity. In contrast, in cells treated with curcumin after LPS stimulation, a less reactive state of the microglia and lower TLR4 immunofluorescence intensity were observed (Figure 7A, B). We next characterized the release of inflammatory mediators in the co-culture supernatants by ELISA. These mediators were all increased dramatically 24 hours after LPS stimulation (*P* < 0.01), but only IL-1β, IL-6 and RANTES were significantly decreased in the curcumin-treated group compared with the vehicle-treated group (*P* < 0.05, Figure 7C, D, G); the differences in TNF-α and MCP-1 between the curcumin-treated group and the vehicle-treated group were not significant following LPS stimulation (*P* > 0.05, Figure 7E, F).

### Curcumin suppressed microglial TLR4/MyD88/NF-κB signaling pathway protein expression *in vitro*

**Figure 8:**
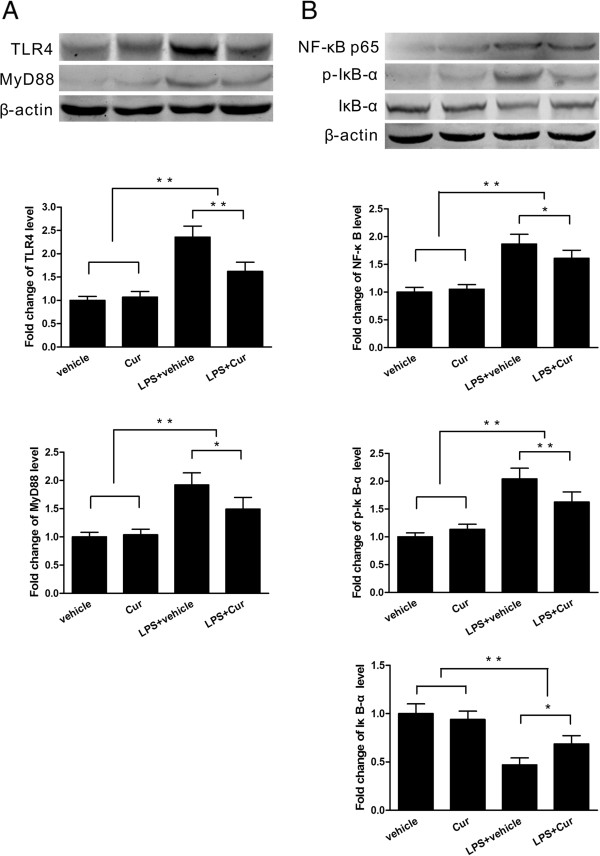
**Curcumin suppressed the expression of proteins in the microglial TLR4/MyD88/NF-**κ**B signaling pathway in co-culture system. (A)** TLR4 and MyD88 protein expression in microglia was significantly lower in the curcumin-treated group than in the vehicle-treated group at 24 hours after lipopolysaccharide (LPS) stimulation. **(B)** NF-κB p65 and p-IκB-α protein expression in microglia was also significantly lower in the curcumin-treated group than in the vehicle-treated group at 24 hours following LPS stimulation. In contrast, IκB-α protein expression was significantly higher in the curcumin-treated group than in the vehicle-treated group after LPS stimulation. Values (mean ± SD) are representative of three independent experiments. *P < 0.05, **P < 0.01.

To further understand the effect of curcumin treatment on TLR4 downstream signaling pathways in microglia, Western blotting was performed to detect the expression of TLR4 and its adapter proteins at 24 hours post-trauma. In the transwell co-cultures of primary neurons and microglia stimulated by LPS, the levels of TLR4 and MyD88 protein expression in microglia were significantly increased compared with those in the two control groups (*P* < 0.01); further, they were significantly decreased in the curcumin-treated group compared with the vehicle-treated group following LPS stimulation (*P* < 0.05, Figure 8A). Similar changes in p-IκB-a and NF-κB p65 were observed. In contrast, IκB-a protein expression was significantly decreased in the two LPS-stimulated groups when compared with the two control groups (*P* < 0.05) and was significantly increased in the curcumin-treated group when compared with the vehicle-treated group following LPS stimulation (*P* < 0.05, 8B).

## Discussion

In this study, we used TLR4^-/-^ mice to investigate the role of TLR4 during the acute stage of TBI and observed reductions in cerebral edema, neurological deficit and neuronal apoptosis at 24 hours post-injury in TLR4^-/-^ mice compared with WT mice. We administered curcumin (100 mg/kg) to WT mice after TBI and observed decreases in microglia/macrophages, inflammatory factor release, neurological deficit and neuronal apoptosis at 24 hours post-injury by inhibiting the TLR4/MyD88/NF-κB signaling cascade. *In vitro*, in a co-culture system of microglial and neuronal cells, LPS administration induced microglial activation and neuronal damage, while 2 μM curcumin could inhibit microglial activation and neuronal apoptosis by suppressing the microglial TLR4 signaling pathway. To our knowledge, we report for the first time that one possible molecular mechanism whereby curcumin attenuates brain injury is the modulation of acute neuroinflammation mediated by the TLR4/MyD88/NF-κB signaling pathway in microglia/macrophages during experimental TBI.

One key factor in secondary brain injury is a complex series of inflammatory responses that is initiated largely through TLRs that possibly interact with endogenous ligands released from damaged cells [32,33]. Furthermore, TLR4, which is widely expressed on the plasma membranes of neural cells, has been demonstrated to play an important role in initiating the cerebral inflammation related to cerebral ischemia-reperfusion injury and intracerebral hemorrhage in TLR4^-/-^ mice [34,35]. Along these lines, neuroinflammatory responses initiated by TLR4 may also be an important factor underlying secondary brain injury after TBI. Indeed, TLR4 protein expression was significantly increased at six hours after brain trauma and remained high at 72 hours compared with the levels observed in the control group in our study, which is consistent with the report of Chen and colleagues [36]. Furthermore, a critical role of TLR4 was demonstrated by our observations that brain water content, neurological deficit score and neuronal death were significantly decreased in TLR4^-/-^ mice in comparison to WT mice suffering a similar severity of head trauma. The neuroprotective effect of TLR4 deficiency in TBI can be partially attributed to the suppression of acute neuroinflammation induced by the inhibition of microglial or peripheral leukocyte activation and the subsequent cytokine release. In Helmy’s clinical study, the release of many cytokines, such as IL-1β, TNF-α and RANTES, peaked at 24 hours post-trauma, and notably, the concentrations of some cytokines (for example, IL-1β, IL-6, MCP-1) were significantly higher in brain tissue than in plasma [37]. In the present study, the upregulation of IL-1β, IL-6, MCP-1 and RANTES in injured brain tissue was dramatically attenuated in injured TLR4^-/-^ mice, although TNF-α was not significantly decreased. Some anti-inflammatory therapies aimed at inhibiting TLR4 activation have displayed neuroprotective effects at 24 hours in animal TBI models [38,39]. Recently, another study of TBI, which showed lower infarct volumes and better outcomes on neurological and behavioral tests in TLR4^-/-^ mice at 24 hours post-injury, has also validated the important role of TLR4 in TBI [10].

We focused on curcumin, which is used as a spice or a pigment, because of its numerous pharmacological activities, very low toxicity and widespread availability. Unfortunately, curcumin exhibits relatively poor oral bioavailability and a short serum half-life (< 45 minutes), which could contribute to its limited therapeutic window (less than one hour post-injury) in head trauma [17,40]. One study reported that in mice, peak plasma concentrations (approximately 1.6 μM) were achieved 15 minutes after the intraperitoneal administration of 100 mg/kg curcumin, followed by brain accumulation within one hour [41]. A much better curcumin bioavailability has been reported in many articles following intraperitoneal injection [41-43]. Thus, we chose an intraperitoneal injection at 15 minutes post-trauma in our study because of the better bioavailability and the limited effective window of curcumin. A 100 mg/kg dose was selected due to its dramatic reduction of TLR4 expression at 24 hours and the relatively low concentration in the three different concentrations administered to mice. In addition, the use of liposomes or nanoparticles may improve drug delivery, overcome bioavailability issues and extend the therapeutic window [40].

In TBI experiments, cognitive disability tested by the Morris water maze has been ameliorated by treatment with curcumin or curcumin derivatives [16,44]; the cognitive protection conferred by curcumin is partially related to the restoration of membrane homeostasis or to normalized levels of brain-derived neurotrophic factor (BDNF) and its downstream effectors of synaptic plasticity (cAMP-response element binding protein, synapsin1). In addition to the cognitive functions described above, locomotor function and brain edema have also been improved with curcumin treatment due to the decrease in the induction of NF-κB and its downstream production of IL-1β in the brain [17]. In mice with intracerebral hemorrhage, the attenuation of hematoma size and neurological injury was also associated with the decreased induction of cytokine expression after curcumin treatment [45]. These findings suggest that immune modulation by curcumin is a promising approach to the treatment of brain injury. Furthermore, TLR4, a critical membrane receptor mediating innate immunity, can induce NF-κB upregulation when it is activated by stimuli [46]. Therefore, for immunomodulation following TBI, TLR4 may be an important target of curcumin. Notably, Youn *et al*. have demonstrated that the TLR4 receptor complex is a molecular target of curcumin and that curcumin can inhibit TLR4 homodimerization [20]. In the present study, the upregulation of TLR4 expression and inflammatory mediator release (IL-1β, TNF-α, MCP-1 and RANTES) was attenuated by curcumin treatment following TBI. Among these mediators, RANTES has been suggested as a significant early marker of severe TBI in critically injured trauma patients [47]. Furthermore, the curcumin-treated group, which exhibited suppressed acute inflammatory responses after TBI, showed ameliorated brain damage, including reduced neurological impairment, brain edema and neuronal and apoptotic cell death. Thus, curcumin could reduce TLR4-mediated post-traumatic acute neuroinflammation, thereby attenuating secondary brain injury. A few studies have also reported that curcumin can attenuate inflammation and subsequent inflammatory injury by inhibiting TLR4 expression in colitis [48], hepatic fibrosis [49] and lung injury [50]. Within the TLR4 signaling pathway, the MyD88-dependent signaling pathway is an important activator of NF-κB and the subsequent regulatory effects of NF-κB signaling [51,52]. In accordance with these reports, the levels of MyD88 and NF-κB were observed to decrease following curcumin administration. These data suggest that the protective effects of curcumin on the brain against excessive inflammatory responses may be mediated by the TLR4/MyD88/NF-κB signaling cascade following TBI.

Microglia, which when activated by exogenous or endogenous ligands produce a number of proinflammatory cytokines implicated in neurotoxicity, are the principal cells involved in the innate immune response in the CNS [53]. In the present study, CD11b-positive cells were reduced in injured brain tissue following curcumin treatment. However, CD11b positivity does not imply that these cells are exclusively microglia; CD11b-positive cells can also include monocytes/macrophages and lymphocytes, which permeated the injured tissue. Peripheral immune infiltration and alterations can also have a significant impact in TBI [54]. However, microglia were our primary interest, and we therefore used a transwell co-culture system with microglia and neurons to further investigate the role of microglia in immunomodulation.

In our experiments *in vitro*, LPS resulted in obvious neuronal damage in both the single-culture and co-culture systems. However, the observation that the damage was more serious in neurons co-cultured with microglia indicates that microglia play an important role in neuronal injury. This was consistent with another report [55], in which low concentrations of LPS induced significant neuronal death in a co-culture system that allowed direct microglial-neuronal contact; however, high concentrations of LPS were necessary to induce neurotoxicity in a transwell system permitting only cell contact-independent communication. Nevertheless, the critical role of microglia in neuronal damage was evident.

Curcumin treatment dramatically alleviated neuronal damage in the co-culture system but had no obvious effect in neuron-only cell culture after LPS stimulation. These results suggest that the protective effect of curcumin on neurons was mediated through microglia. Similarly, a previous study showed that curcumin protected dopaminergic neurons from MPP^+^-induced neurotoxicity in rat mid-brain neuron-glia co-cultures and that the protective effect of curcumin disappeared in microglia-depleted cultures [56]. Furthermore, curcumin had an inhibitory effect on microglial migration in a BV-2 cell scratch model and transwell migration model [57]. The TLR-induced activation of microglia and the release of proinflammatory molecules are responsible for neurotoxic processes in the course of some CNS diseases [58,59]. In the present transwell co-culture system, curcumin treatment inhibited TLR4 expression in microglia, the morphological activation of microglia and inflammatory mediator release following LPS stimulation, and these findings are consistent with the observed attenuated neuronal damage. These *in vitro* observations suggest that the inhibition of microglial TLR4 may be one reason underlying the suppression of neuroinflammation and the protection of neurons following curcumin treatment. In regard to the TLR4 pathway, one study reported that a functional TLR4/MyD88 cascade in microglia was essential for neuronal injury induced by HSP60 via the co-culture of WT neurons with MyD88^-/-^ or Lps^d^ microglia (hyporesponsiveness to LPS as a consequence of a point mutation rendering the cytosolic domain of TLR4 incapable of signal transduction) [60]. In another study of rat vascular smooth muscle cells (VSMCs), curcumin suppressed the LPS-induced overexpression of inflammatory mediators in VSMCs by inhibiting the TLR4/NF-κB pathway [24]. In our co-culture system including primary WT neurons and microglial cells, the protein levels of TLR4 and downstream molecules (MyD88, p-IκB-α and NF-κB) in microglia were increased by LPS, and curcumin attenuated the upregulation of these molecules in the TLR4 pathway. These data further indicate that curcumin regulates a complex series of inflammatory responses contributing to neuronal damage, in part through the microglial TLR4/MyD88/NF-κB signaling pathway.

Interestingly, in contrast to LPS administration after brain injury, LPS preconditioning protected the brain from ischemic injury through the redirection of TLR4 signaling, including the suppression of NF-κB activity, enhancement of interferon regulatory factor 3 (IRF3) activity and an increase in anti-inflammatory/type I interferon gene expression [61,62]. In TBI, LPS preconditioning has also been shown to confer a long-lasting neuroprotective effect associated with the modulation of microglia/macrophage activity and cytokine production [63]. In a study of cold-induced cortical injury, microglial activation in response to peripheral LPS preconditioning largely depended on nonhematogenous TLR4 receptors, and these activated microglia resulted in reduced inhibitory axosomatic synapses for neuroprotection [64]. The role of the microglial TLR4 signaling pathway in this type of neuroprotection warrants further investigation. Notably, immune modulation by curcumin is robust, and the TLR4 signaling pathway may not be an exclusive mechanism through which curcumin modulates neuroinflammation and contributes to secondary brain injury. However, modulation of the TLR4 pathway was undeniably critical for the neuroprotection mediated by curcumin post-TBI.

In conclusion, our findings demonstrated a critical role for TLR4 of microglia/macrophages in acute neuroinflammation following TBI. Post-injury treatment with curcumin attenuated TLR4-mediated acute activation of microglia/macrophages, proinflammatory mediator release and neuronal apoptosis in the injured brain tissue via inhibition of the MyD88/NF-κB signaling cascade, and this may be an important mechanism through which curcumin improves outcome following TBI. All the data support modulation of the TLR4/MyD88/NF-κB signaling pathway in microglia/macrophages as a potential therapeutic target in TBI and suggest that curcumin should be considered a candidate for clinical trials in TBI.

## Abbreviations

BDNF: brain-derived neurotrophic factor; DAMP: damage-associated molecular pattern; DAPI: 4',6-diamidino-2-phenylindole; DMSO: dimethyl sulfoxide; DMEM: Dulbecco’s modified Eagle’s medium; ELISA: Enzyme-Linked Immunosorbent Assay; FJB: Fluoro-Jade B; IRF3: interferon regulatory factor 3; HBSS: Hank’s balanced saline solution; LPS: lipopolysaccharide; MCP: monocyte chemoattractant protein; MTT: 3-(4,5-dimethylthiazol-2-yl)-2,5- diphenyltetrazolium bromide; MyD88: myeloid differentiation factor 88; NF-κB: nuclear factor-kappa B; PAMP: pathogen-associated molecular pattern; PBS: phosphate-buffered saline; PRR: pathogen-recognition receptors; RANTES: regulated upon activation, normal T cell expressed and secreted; SDS-PAGE: sodium dodecyl sulfate polyacrylamidegel electrophoresis; TBI: traumatic brain injury; TdT: terminal deoxynucleotidyltransferase; TLR: Toll-like receptor; TNF-α: tumor necrosis factor alpha; VSMC: vascular smooth muscle cell; WT: wild-type.

## Competing interests

The authors declare that they have no competing interests.

## Authors’ contributions

This study was based on the original idea of JKL and HF. HTZ and CB carried out the molecular biology and morphological studies and drafted the manuscript. XX and FC carried out the behavioral studies. JCY and CSW performed data analyses. JKL and HTZ were responsible for supervising all experiments, data analyses and the drafting of the manuscript. WHC read and revised the manuscript. All authors read and approved the final manuscript.
